# Decreased 16:0/20:4-phosphatidylinositol level in the post-mortem prefrontal cortex of elderly patients with schizophrenia

**DOI:** 10.1038/srep45050

**Published:** 2017-03-23

**Authors:** Junya Matsumoto, Hiroki Nakanishi, Yasuto Kunii, Yuki Sugiura, Dai Yuki, Akira Wada, Mizuki Hino, Shin-Ichi Niwa, Takeshi Kondo, Michihiko Waki, Takahiro Hayasaka, Noritaka Masaki, Hiroyasu Akatsu, Yoshio Hashizume, Sakon Yamamoto, Shinji Sato, Takehiko Sasaki, Mitsutoshi Setou, Hirooki Yabe

**Affiliations:** 1 Department of Neuropsychiatry, School of Medicine, Fukushima Medical University, 1 Hikarigaoka, Fukushima, Fukushima 960-1295, Japan; 2 Research Center for Biosignal, Akita University, 1-1-1 Hondo, Akita, Akita 010-8543, Japan; 3 Akita Lipid Technologies, LLC.,1-2, Nukazuka, Yanagida, Akita, 010-0825, Japan; 4 Department of Psychiatry, Aizu Medical Center, Fukushima Medical University, 21-2 Maeda, Yazawa Kawahigashimachi, Aizuwakamatsu, Fukushima 969-3492, Japan; 5 Department of Cellular and Molecular Anatomy, Hamamatsu University School of Medicine, 1-20-1, Handayama, Higashi-ku, Hamamatsu, Shizuoka 431-3192, Japan; 6 Department of Neuropsychiatry, The University of Tokyo Hospital, 7-3-1, Hongo, Bunkyo-ku, Tokyo 113-8655, Japan; 7 Choju Medical Institute, Fukushimura Hospital, 19-14 Yamanaka, Noyori-cho, Toyohashi, Aichi 441-8124, Japan; 8 Department of Community-based Medical Education/Department of Community-based Medicine, Nagoya City University Graduate School of Medical Science, 1, Kawasumi, Mizuho-cho, Mizuho-ku, Nagoya, Aichi 467-8601, Japan; 9 Quests Research Institute, Otsuka Pharmaceutical Co. Ltd., 463-10 Kagasuno, Kawauchi-cho, Tokushima, Tokushima 771-0192, Japan; 10 Department of Medical Biology Graduate School of Medicine, Akita University, 1-1-1 Hondo, Akita, Tokushima 010-8543, Japan; 11 International Mass Imaging Center, Hamamatsu University School of Medicine, 1-20-1 Handayama, Higashi-ku, Hamamatsu, Shizuoka 431-3192, Japan; 12 Preeminent Medical Photonics Education & Research Center, Hamamatsu University School of Medicine, 1-20-1 Handayama, Higashi-ku, Hamamatsu, Shizuoka 431-3192, Japan; 13 Department of Anatomy, The university of Hong Kong, 6/F, William MW Mong Block 21 Sassoon Road, Pokfulam, Hong Kong SAR, China; 14 Division of Neural Systematics, National Institute for Physiological Sciences, 38 Nishigonaka Myodaiji, Okazaki, Aichi, 444-8585, Japan; 15 Riken Center for Molecular Imaging Science, 6-7-3 Minatojima-minamimachi, Chuo-ku, Kobe, Hyogo 650-0047, Japan

## Abstract

The etiology of schizophrenia includes phospholipid abnormalities. Phospholipids are bioactive substances essential for brain function. To analyze differences in the quantity and types of phospholipids present in the brain tissue of patients with schizophrenia, we performed a global analysis of phospholipids in multiple brain samples using liquid chromatography electrospray ionization mass/mass spectrometry (LC-ESI/MS/MS) and imaging mass spectrometry (IMS). We found significantly decreased 16:0/20:4-phosphatidylinositol (PI) levels in the prefrontal cortex (PFC) in the brains from patients with schizophrenia in the LC-ESI/MS/MS, and that the 16:0/20:4-PI in grey matter was most prominently diminished according to the IMS experiments. Previous reports investigating PI pathology of schizophrenia did not identify differences in the *sn*-1 and *sn*-2 fatty acyl chains. This study is the first to clear the fatty acid composition of PI in brains from patients with schizophrenia. Alteration in the characteristic fatty acid composition of PI may also affect neuronal function, and could play a role in the etiology of schizophrenia. Although further studies are necessary to understand the role of reduced 16:0/20:4-PI levels within the prefrontal cortex in the etiology of schizophrenia, our results provide insight into the development of a novel therapy for the clinical treatment of schizophrenia.

Schizophrenia is a psychiatric disorder characterized by thought disturbance; its etiology is known to include structural abnormalities related to faulty neurodevelopment. Such gross abnormalities could be related to neuronal-level abnormalities; indeed, one class of molecule, the phospholipid, has been linked to structural abnormalities in schizophrenia^1^. The lipid bilayer of cell membranes is mainly composed of phospholipids, which are broken down by several classes of phospholipases for use as second messengers in signal transduction pathways in neural and glial cells^2^. In fact, phospholipids make up approximately 60% of the brain’s dry weight^3^,^4^, and 20–30 different types of fatty acids are attached to the phosphate backbones. Thus, there is an abundance of diverse phospholipids in the cell membrane, including particularly important variants such as phosphatidylcholine (PC), phosphatidylethanolamine (PE), phosphatidylserine (PS), and phosphatidylinositol (PI)^1^,^5^. Phospholipids are bioactive substances essential for brain function, and previous works indicated a link between them and the etiology of schizophrenia^1^,^5^. These studies indicated alterations of the phospholipids^1^,^5^, but methodological constraints prevented their reporting on the details of the affected phospholipids, such as the specific fatty acid compositions/combinations. The new methodological approaches allowing investigation of the precise profiles of phospholipids are essential for a comprehensive examination of the relationship between phospholipids and the etiology of schizophrenia.

To determine this relationship, research on the phospholipids themselves is needed. A number of post-mortem studies using brain tissue from patients with schizophrenia have in fact been performed. However, despite having used advanced methodologies including magnetic resonance spectroscopy (MRS), high performance liquid chromatography, and gas chromatography, the precise details regarding the composition of fatty acid residues in the brain phospholipids, and their specific combinations within the phospholipid molecules themselves, were unable to be established^6^,^7^,^8^. Some previous research suggested that prostaglandins, synthesized from fatty acids obtained from phospholipase activity or food, might be associated with schizophrenia^9^,^10^. Other studies showed decreased polyunsaturated fatty acids in red blood cell membranes from patients with schizophrenia; the appropriateness of this finding was also supported by meta-analysis studies^11^,^12^. In contrast, a Cochrane review^13^ indicated that the clinical effects polyunsaturated fatty acid administration for schizophrenia have been inconsistent. Hence, fatty acid disturbances might not be central to the etiology of schizophrenia. Additionally, elevated maternal docosahexaenoic acid (DHA) levels during pregnancy were found to be associated with schizophrenia risk, and polyunsaturated fatty acids in schizophrenia showed a bimodal distribution^14^, indicating that analyzing fatty acids alone cannot reveal lipid abnormalities present in schizophrenia. Thus, the best analyses for identifying the role of lipid abnormalities in the etiology of schizophrenia must concentrate on the phospholipids, which are the main source of the lipid signaling related fatty acids in brain tissue^15^. Furthermore, while magnetic resonance spectroscopy can be applied to the study of living patients’ brains^16^, it cannot distinguish the different specific types of fatty acid residues, so the above-mentioned phospholipid analyses require the use of post-mortem tissue.

The present study investigates phospholipid expression in the prefrontal cortex (PFC) and superior temporal gyrus (STG). Dysfunction of the PFC in schizophrenia is associated with the cognitive impairment that is central to schizophrenia^17^. Some studies indicate that Brodmann area 10 (BA10) in the PFC is involved in multitasking, social cognition, working memory, and episodic memory^18^,^19^; thus, we focused on the PFC and extracted lipids from BA10 of post-mortem brains. In addition, some studies suggest that dysfunction in the STG is associated with the auditory hallucinations^20^,^21^ that are a typical symptom of schizophrenia. To further investigate the association between lipid alterations and clinical features, we also analyzed Brodmann area 22 (BA22), the main area in the STG.

In the present study, we used liquid chromatography electrospray ionization mass/mass spectrometry (LC-ESI/MS/MS) to reveal significantly decreased 16:0/20:4-PI and 18:0/22:6-PS levels in the PFC of brains from patients with schizophrenia. In the imaging mass spectrometry (IMS) experiments, we also found that 16:0/20:4-PI and 18:0/22:6-PS in the gray matter were prominently diminished in patients with schizophrenia. Previous reports investigating the PI abnormality in schizophrenia did not identify differences in the *sn*-1 and *sn*-2 fatty acyl chains. The present study is the first to identify the fatty acid composition of PI in brains from patients with schizophrenia. LC-ESI/MS/MS allowed us to quantitatively define the fatty acid residue compositions of the phospholipids, while IMS provided clear, two-dimensional images of the spatial distribution of hundreds of phospholipids in a single measurement without the need for tissue homogenization. The combination of microscopy with high-resolution matrix-assisted laser desorption/ionization-imaging mass spectrometry (MALDI-IMS) promises to be a useful tool in post-mortem brain studies of schizophrenia^22^, given its capacity for unprecedented detail and ability to create a precise profile of the phospholipids in post-mortem brains.

## Results

### LC-ESI/MS/MS analysis revealed acyl chain-specific alterations of phospholipids in the PFC of patients with schizophrenia

First, we extracted lipids from post-mortem brain samples from patients with schizophrenia or control samples and performed LC-MS analysis. Demographic information for all subjects is indicated in Table 1. Our measurement method was optimized for quantitative analysis of minor class lipids, resulted in irrelevant for high throughput analyses and small number of samples compared to common metabolome studies. However, we conclude that the samples size is appropriate based on the effect size (Cohen’s f).

**Figure 1:**
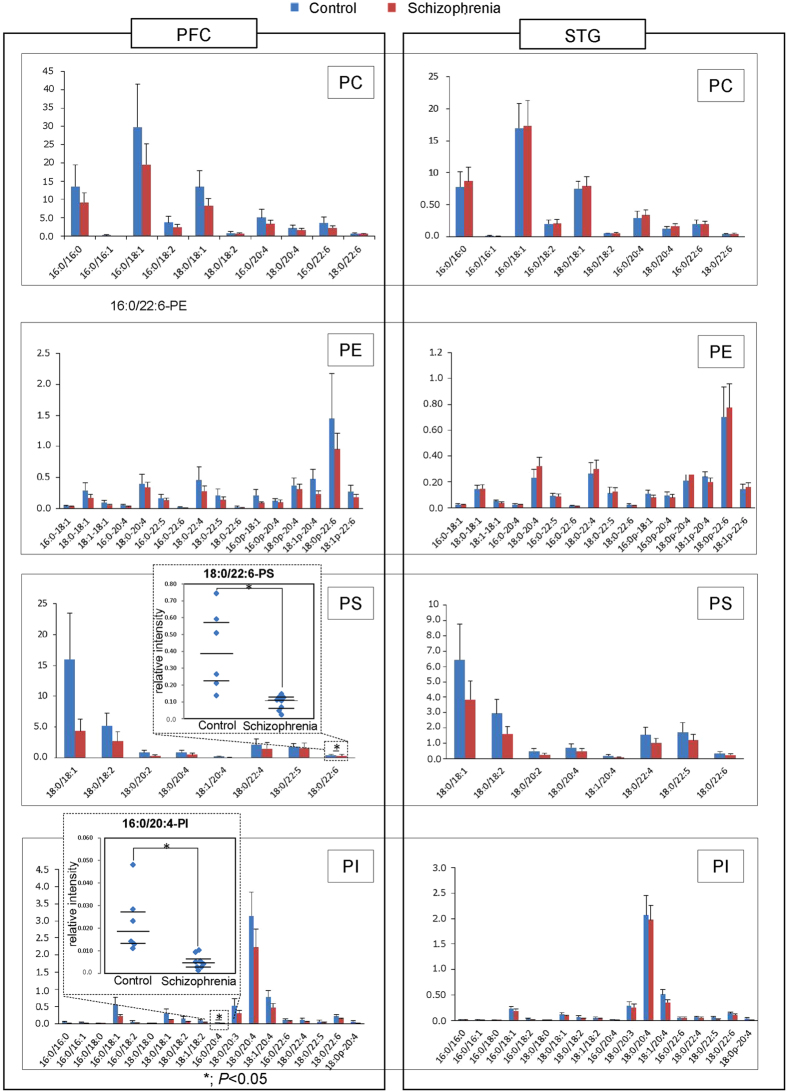
Quantification and analysis of phospholipids in brain tissue samples. The amounts of phosphatidylcholine (PC), phosphatidylethanolamine (PE), phosphatidylserine (PS), and phosphatidylinositol (PI) in the prefrontal cortex (PFC) and superior temporal gyrus (STG) are presented in the bar charts, with the y-axis indicating the relative intensity of each phospholipid as quantified by LC-ESI/MS/MS in the PFC and the STG gray matter. The leftmost charts indicate the relative intensity of PFC gray matter, and the rightmost charts represent relative intensity of the STG. A scatterplots that is specific for 16:0/20:4-PI and 18:0/22:6-PS are embedded in the PI and PS bar charts, respectively.

**Figure 2:**
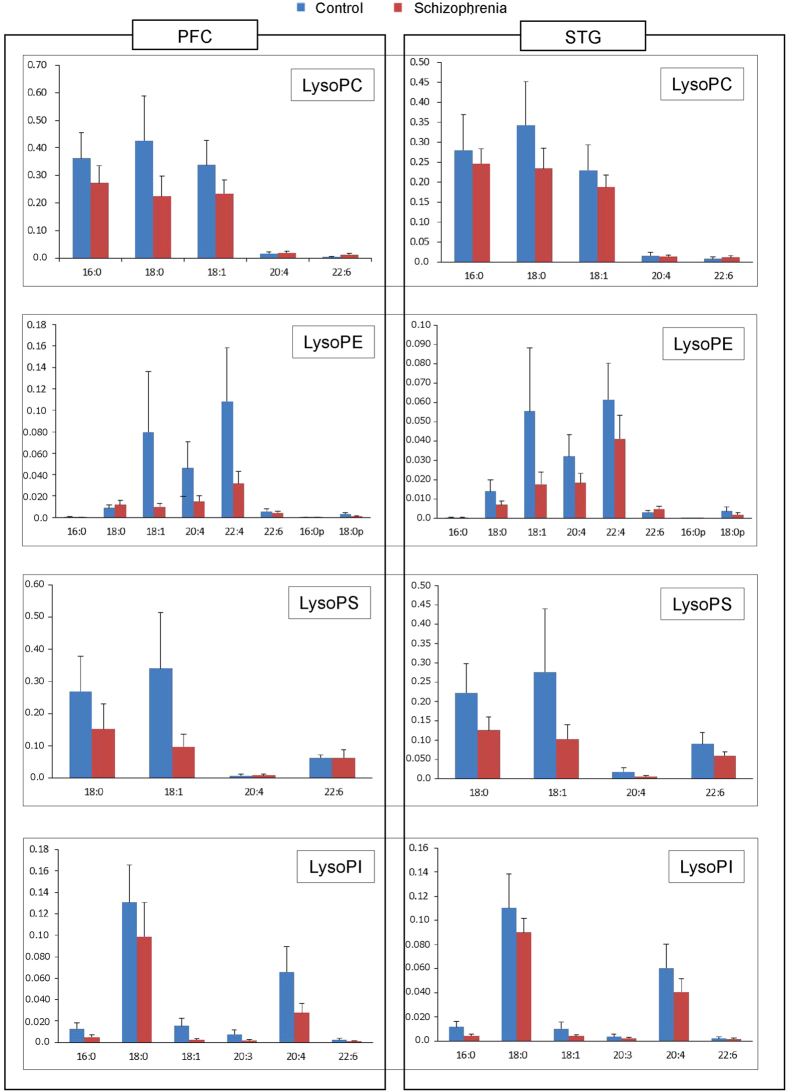
Quantification and analysis of lysophospholipids in brain tissue samples. The amounts of lysophosphatidylcholine (LysoPC), lysophosphatidylethanolamine (LysoPE), lysophosphatidylserine (LysoPS), and lysophosphatidylinositol (LysoPI) in the prefrontal cortex (PFC) and superior temporal gyrus (STG) are presented in the bar charts, such that the y-axis indicates the relative intensity of each phospholipid as quantified by LC-ESI/MS/MS in the PFC and the STG gray matter. The leftmost charts indicate the relative intensity of PFC gray matter, and the rightmost charts represent relative intensity of the STG.

Figure 1 shows the amounts of PC, PE, PS, and PI in the PFC and STG as assessed by LC-ESI/MS/MS. Each bar chart indicates the relative intensity of each phospholipid species in the PFC and the STG gray matter, in a vertical direction, and quantified by LC-ESI/MS/MS. LC-ESI/MS/MS successfully evaluated the amount of individual phospholipids according to their acyl chains in the *sn*-1 and *sn*-2 positions. In terms of quantity, phospholipids containing 16:0/18:1 were the most abundant of the PC family, followed by 16:0/16:0 and 18:0/18:1. In the PE family, 18:0p-22:6 was the most abundant, while in the PS family, 18:0/18:1 was the most abundant. Finally, in the PI family, 18:0/20:4 was the most abundant. The Fig. 2 shows the amounts of lysophosphatidylcholine (LysoPC), lysophosphatidylethanolamine (LysoPE), lysophosphatidylserine (LysoPS), and lysophosphatidylinositol (LysoPI). Lysophospholipids containing 18:0, 18:1, and 16:0 were the most abundant of the LysoPC family, while in the LysoPE family, 22:4, 20:4, 18:1, and 18:0 were abundant. In the LysoPS family, 18:0, 18:1 were abundant, and in the LysoPI family, 18:0 and 20:4 were abundant.

Next, we investigated the quantity of phospholipids in patients with schizophrenia and compared that with the control group. We performed a Shapiro-Wilk W test of normality to reveal that this data were normally distributed; therefore, we choose to subsequently perform a parametric analysis. We found that PI containing 16:0/20:4 [16:0/20:4-PI, analysis of covariance (ANCOVA): *F* (1,9) = 7.01, *P* = 0.027, Effect size f = 0.95], and PS containing 18:0/22:6 [18:0/22:6-PS, ANCOVA: *F* (1,9) = 7.13, *P* = 0.026, Effect size f = 0.96] were significantly lower in the schizophrenia group than the control group in the PFC (Fig. 1). Except for the decreases in 16:0/20:4-PI and 18:0/22:6-PS in the PFC, all other observed patterns of PI and PS-based fatty acids were not significantly different between groups. Additionally, among the arachidonoyl acyl chain-containing phospholipids and docosahexaenoyl chain-containing phospholipids in the PFC, only 16:0/20:4-PI and 18:0/22:6-PS showed a reduced level, indicating the alterations in schizophrenia have a highly specificity in terms of both of phospholipid class and acyl chains.

### MALDI-IMS analysis revealed a reduction of 16:0/20:4-PI and 18:0/22:6-PS in the gray matter, but not the white matter of patients with schizophrenia

**Figure 3:**
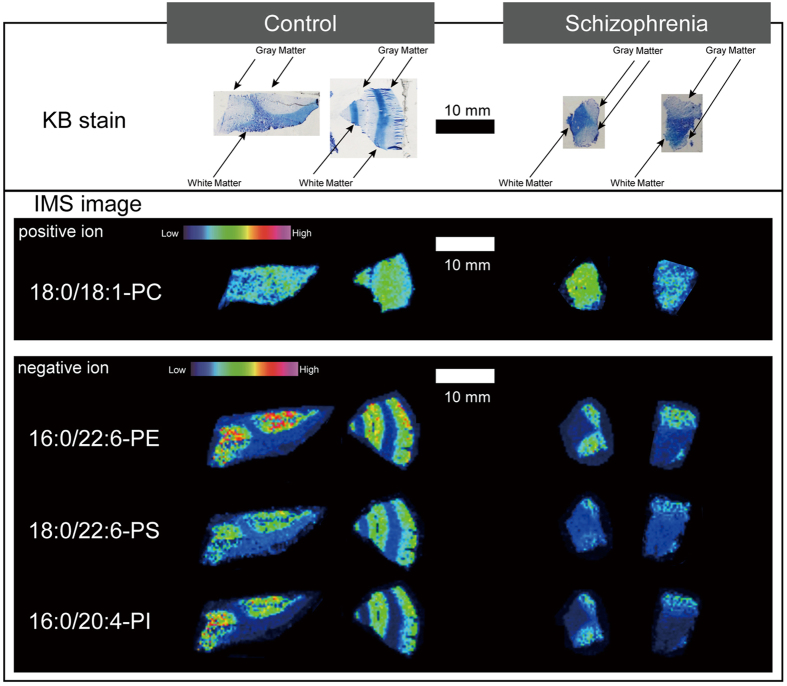
Representative post-mortem prefrontal cortex (PFC) sample images. In the upper row, Kluver-Barrera (KB)-stained PFC sample images differentiate brain regions consisting of predominately gray matter or white matter. The sample images in the second row are MALDI-IMS images of representative phospholipids matched with their KB-stained images above.

Because the arachidonic acid-containing PI molecules are important sources for regulating downstream signaling in the brain^23^,^24^, we further investigated spatial changes of 16:0/20:4-PI and 18:0/22:6-PS in PFC samples from patients with schizophrenia. As Fig. 3 shows, MALDI-IMS analysis demonstrated heterogeneity in the distribution of 16:0/20:4-PI and 18:0/22:6-PS within the schizophrenia and control groups, and specifically in the gray matter. To differentiate between gray and white matter regions, Kluver-Barrera (KB)–stained (i.e., Luxol Fast Blue) images of the regions were utilized. In both groups, the intensity of 16:0/20:4-PI and 18:0/22:6-PS in the gray matter was higher than that in white matter (Fig. 3). Brains from patients with schizophrenia demonstrated much a lower intensity of 16:0/22:4-PI and 18:0/22:6-PS in the gray matter than brains from control subjects. The intensities of other phospholipid species, such as 18:0/18:1-PC, and 16:0/22:6-PE, did not show clear differences in the gray matter.

### Correlations between clinical profiles and 16:0/20:4-PI and 18:0/22:6-PS alterations

The specific reduction of phospholipids prompted us to assess the correlations between clinical profiles and the quantity of 16:0/20:4-PI and 18:0/22:6-PS in the PFC across patients with schizophrenia. As shown in Table 2, no correlation was observed between the duration of illness (DOI), and the estimated total dosage of prescribed neuroleptics and anticholinergics. There was no significant difference in brain pH between the schizophrenia and control groups (t test; *P* = 0.22), and we assessed the relationship between phospholipid levels and brain pH in the two groups. Brain pH was not correlated with 16:0/20:4-PI (*P* = 0.33, r = 0.40) and 18:0/22:6-PS (*P* = 0.87, r = 0.068) in patients with schizophrenia. Furthermore, brain pH in all subjects was not correlated with 16:0/20:4-PI (*P* = 0.86, r = −0.054) and 18:0/22:6-PS (*P* = 0.53, r = −0.18).

## Discussion

In this study, we found significantly lower 16:0/20:4-PI and 18:0/22:6-PS levels in the PFC in samples from patients with schizophrenia, using quantitative analyses with LC-ESI/MS/MS. We further assessed spatial distributions of phospholipids by MALDI-IMS analysis, and demonstrated a distinct difference in the distribution of 16:0/20:4-PI and 18:0/22:6-PS between the gray matter and white matter in the human PFC. The MALDI-IMS images showed that 16:0/20:4-PI and 18:0/22:6-PS in the gray matter of brains from patients with schizophrenia was most prominently diminished.

Most studies about lipid analysis using post-mortem brains from patients with schizophrenia examined only fatty acids, and not phospholipids^25^,^26^,^27^. One study investigated the combination of fatty acyl chains and phospholipids by using LC/MS in the post-mortem brains from patients with schizophrenia, but did not study PI specifically^28^. Using MRS, they could determine PI or PS levels, but not their fatty acid composition^6^,^7^. Though useful *in vivo*, MRS lacks the necessary specificity in those conditions as well^16^.

Previous studies investigating PI in post-mortem brains from patients with schizophrenia^29^,^30^ could neither distinguish the combination of the fatty acyl chains in the *sn*-1 and *sn*-2 positions, nor compare diagnostic differences in the fatty acids when combined in the *sn*-1 and *sn*-2 positions. Thus, no previous study identified the specific fatty acid combinations within PI.

The LC-ESI/MS/MS methods can identify each fatty acid combination in the phospholipids in both the *sn*-1 and *sn*-2 positions by their ester bonds, though gas chromatography cannot distinguish the individual fatty acyl chains. Thus, we could identify both the phospholipids and the fatty acyl chains in the *sn*-1 and *sn*-2 positions in the post-mortem brains from patients with schizophrenia, unlike previous post-mortem brain studies. In addition, most of these analytical techniques had to use homogenized samples, so structural information was lost. Hence, such studies are limited in their ability to specifically quantify gray matter or white matter, as opposed to homogenates of both types of tissue. Our quantitative analysis data were verified in terms of the type of brain tissue by checking against the IMS-based results. When there is a need to elucidate the precise phospholipid species, as well as the two-dimensional structural information of the brain tissue samples, only IMS is capable. For these reasons, we found the combination of LC-ESI/MS/MS and IMS to be most useful.

In the present study, we found 16:0/20:4-PI and 18:0/22:6-PS reductions in BA10, one of the regions in the PFC, but not in the STG. It has been repeatedly reported that dysfunction of the PFC in schizophrenia is closely associated with the cognitive impairment that is central to schizophrenia^17^. Furthermore, some studies have indicated that BA10 is involved in multitasking, social cognition, working memory, and episodic memory^18^,^19^. In contrast, dysfunction in the STG is associated with auditory hallucinations^20^,^21^. Thus, we feel it reasonable to hypothesize that the disturbance in 16:0/20:4-PI or 18:0/22:6-PS appear to be relevant to PFC dysfunction and some cognitive impairment, but not to the auditory symptoms originating in the STG. Further study is needed to verify the association between disturbed PI metabolism and the cognitive impairment in patients with schizophrenia.

IMS analysis further clarified that 16:0/20:4-PI and 18:0/22:6-PS are mainly distributed in the gray matter, and patients with schizophrenia showed lower levels of these lipids. The white matter is comprised of abundant axons and myelin sheaths with many phospholipids that form lipid bilayer membranes, while the gray matter contains relatively abundant neuronal cell bodies, dendrites, and synaptic structures. Considering the observation that a lower number of dendritic spines are found within the DLPFC in schizophrenia^31^, the lower levels of 16:0/20:4-PI and 18:0/22:6-PS may be associated with spine loss. Additionally, PI-related signal pathways play a pivotal role in the regulation of spine plasticity^32^. Therefore, our results indicate that the lower phospholipid levels are related to a deficiency in the regulation of synaptic plasticity, which would lead to cognitive impairment.

PI seems to be the most important phospholipid, given its role as the precursor of inositol trisphosphate (IP3) and diacylglycerol (DAG), two critical second messengers^23^,^24^ whose functionalities are affected by the fatty acid combinations from which they are derived. DAGs produced from phosphatidylinositol 4,5-bisphosphate (PI(4,5)P2) by phospholipase C (PLC) contain (unsaturated) arachidonic acid and activate protein kinase C (PKC), whereas DAGs containing saturated fatty acids do not effectively activate PKC^33^,^34^. Notably, PLCβ1 knockout mice demonstrated schizophrenia-like behaviors with dysregulated adult hippocampal neurogenesis^35^, and deletions of PLCβ1 were seen in the orbitofrontal cortex of patients with schizophrenia^36^. Thus, we hypothesize that the lower 16:0/20:4-PI level is related to the etiology of schizophrenia through a dysfunction of the DAG- and PLC/PKC-mediated signaling pathway. Remarkably, lithium carbonate, which has been used for augmentation therapy of schizophrenia^37^, prevents the synthesis of phosphatidylinositol bisphosphate (PIP2) and subsequent generation of IP3 and DAG^38^. Therefore, impaired 16:0/20:4 levels that prevent PIP2 synthesis might be related to the etiology of schizophrenia, and appears to be a useful target for novel antipsychotics. In addition to IP3/DAG, the arachidonic acid-containing PI could be the precursor of 2-arachidonoylglycerol (2-AG), an endocannabinoid putatively related to schizophrenia^39^,^40^. The reduction of arachidonic acid-containing PI may reflect an increased level of 2-AG in schizophrenia^40^. These findings suggest that cannabinoid-related psychosis appears to be associated with lower 16:0/20:4-PI. The phosphoinositides formed from PI have various bioactivities *in vivo*, including involvement in the synthesis of the aforementioned second messengers. Phosphoinositide 3-kinase (PI3K) is an important PI-related kinase that affects the PI3K-Protein kinase B (Akt) signaling pathway, by converting PIP2 to phosphatidylinositol trisphosphate (PIP3). Multiple previous studies have indicated a relationship of single-nucleotide polymorphisms (SNPs) in PI3K family members and schizophrenia^41^,^42^,^43^,^44^. Therefore, it is possible that lower 16:0/20:4-PI levels affect PI3K-Akt signaling, and dysfunction of this pathway may be a mechanism of schizophrenia pathology. In the metabolic pathways of PI, several enzymes have arachidonoyl specificity, leading to the enrichment of arachidonic acid-containing PI^45^. This acyl chain specificity indicates that loss of 16:0/20:4-PI could have more of an affect on the metabolism and function of PI-related species than changes in non arachidonic acid-containing PIs. The fact that the specific fatty acyl composition of PIs has been repeatedly found to strongly affect neuronal function^46^,^47^,^48^,^49^,^50^,^51^ could be related to schizophrenia via lower 16:0/20:4-PI levels, as mentioned above.

Incidentally, PS has been evaluated in the post-mortem brains of patients with schizophrenia. Notably, one study^28^ investigated the combination of fatty acyl chains and PS in post-mortem hippocampus of patients with schizophrenia. PS is relatively easier to investigate than PI because of the small amount of PI in the brain. The results of the study on PS species are different from ours, indicating that the combination of PS species identified depends on the function of the brain area investigated in patients with schizophrenia. Some studies have investigated the role of PS in brain metabolism and others functions^52^; however, further study is needed to understand the relationship between schizophrenia and PS function.

There are several limitations to the present study. Post-mortem brain studies generally should be evaluated with special caution because it is difficult to control disease-related factors. For example, medication or other disease-related factors, or both, may confound the results by affecting the level of PLs or fatty acid residues. Although we did not detect any effect of antipsychotic drugs on 16:0/20:4-PI and 18:0/22:6-PS levels in this post-mortem study, further studies using animal models are warranted to examine the effects of chronic administration of antipsychotics on amounts of these lipids in the PFC. Secondly, our study population was small, and control samples were from patients older than the patients with schizophrenia. However, we found no correlations between either age or post mortem interval (PMI) and 16:0/20:4-PI and 18:0/22:6-PS levels in the PFC. That said, the findings must be confirmed via post-mortem brain investigation in a younger, larger, and more closely matched cohort. Another limitation involves the control subjects. We only estimated that the control subjects did not have a psychiatric disease based on their medical records. The controls were not previously administered a structured interview or a psychiatric examination.

In summary, our results show that the amount of 16:0/20:4-phosphatidylinositol was lower in post-mortem brain samples from patients with schizophrenia than in those from control subjects, and this difference was selectively observed in the gray matter of the PFC. This change may reflect important molecular mechanisms involved in the development of the hypofrontality that is prominent in schizophrenia. Additionally, in the case of peripheral tissues such as blood cells reflect this difference in 16:0/20:4-PI levels, measurement of 16:0/20:4-PI levels in those tissues could represent a new diagnostic test for schizophrenia. Should low 16:0/20:4-PI levels be causative in the etiology of schizophrenia, this finding may also be insightful toward the development of a new therapeutic avenue for treating schizophrenia. Future studies should identify the factors that reduce 16:0/20:4 PI levels, and how this reduction contributes to the etiology of schizophrenia.

## Methods and Materials

### Human brain tissue samples

Post-mortem brain samples from BA10 in the PFC and BA22 in the STG were obtained from patients who had been diagnosed with schizophrenia from the Post-mortem Brain Bank of Fukushima for Psychiatric Research (Fukushima, Japan). Control samples were obtained from the Choju Medical Institute, Fukushimura Hospital. This research, including the use of post-mortem human brain tissue, was approved by the Ethics Committee of Fukushima Medical University and Fukushimura Hospital, and complied with the Declaration of Helsinki. All procedures were carried out with the informed written consent of the next of kin. All patients diagnosed with schizophrenia had fulfilled the diagnostic criteria established by the American Psychiatric Association (Diagnostic and Statistical Manual of Mental Disorders: DSM-IV). For quantitative analyses, gray matter tissue samples from PFC and STG were analyzed by LC-ESI/MS/MS analysis. For IMS, tissue blocks frozen to −18 °C were sectioned at 8-μm thickness using a cryostat (CM1950; Leica, Germany) as described previously^53^,^54^. Though tissue blocks were held in place by Optimum Cutting Temperature (OCT) polymer, they were not embedded in it to prevent residual polymer on the tissue slices from degrading the mass spectra^54^. The frozen thin sections were thaw-mounted on steel plates (MTP 384 target ground steel T F; Bruker Daltonics) for single MS imaging, and indium-tin-oxide (ITO)-coated glass slides (Bruker Daltonics, Leipzig, Germany) for MS/MS imaging.

### Lipid extraction for analysis of phospholipid molecular species

An internal standard, 14:0/14:0-PC (Avanti), was mixed with the frozen tissue sample, and the lipids were extracted using the Bligh-Dyer method. Briefly, samples were homogenized with 1.5 mL of chloroform/methanol (1/2 v/v), and 0.4 mL water added to the resultant homogenates. The solutions were left for 10 min at room temperature, mixed with 0.5 mL of chloroform and 0.5 mL of water, and allowed to separate into two phases. The lower phase of each sample (total lipid extract) was transferred to a new tube and vacuum dried with an evaporator. Extracted lipids were dissolved in chloroform/methanol (1/2 v/v) and stored at −30 °C. Before the LC-ESI/MS/MS analysis, aliquots of the stored samples were dried under a gentle stream of nitrogen gas and re-dissolved in methanol.

### Reverse phase chromatography

An UltiMate 3000 LC system (Thermo-Fisher Scientific) equipped with an HTC PAL autosampler (CTC Analytics) was used. An aliquot (10 μL) of the lipid extract was injected, and the lipids separated on a Waters X-Bridge C18 column (3.5 μm, 150 mm × 1.0 mm i.d.) at room temperature (25 °C) using the following gradient: Mobile phase A (isopropanol/methanol/water (5/1/4 v/v/v) supplemented with 5 mM ammonium formate and 0.05% ammonium hydroxide); mobile phase B (isopropanol supplemented with 5 mM ammonium formate and 0.05% ammonium hydroxide) at ratios of 70%/30% (0 min), 50%/50% (0–2 min), 20%/80% (2–13 min), 5%/95% (13–15 min), 5%/95% (15–30 min), 95%/5% (30–31 min), 95%/5% (31–35 min) and 70%/30% (35–45 min). Flow rate was set to 20 μL/min.

### LC-ESI/MS/MS analysis

ESI/MS/MS analysis was performed on a triple-stage quadrupole mass spectrometer (TSQ-Vantage from Thermo-Fisher Scientific) connected to the LC system. Phospholipid (PL) species were measured by selected reaction monitoring (SRM) in negative ion mode. The characteristic fragments of individual PLs were detected by product ion scan (MS/MS mode). Conditions for the detection of individual PLs in SRM mode are summarized in Table 3. Data are expressed as means ± standard deviation (SD). Statistical differences between the schizophrenia and control samples were assessed using an ANCOVA for comparisons between groups (schizophrenia versus control); diagnosis were set as independent variables, and age, PMI, and freezer storage time were set as covariates using Statistica Ver. 12.6 (Statsoft Inc., Tulsa, OK USA). A *P* < 0.05 was considered significant. Pearson’s product–moment correlation coefficient was used to analyze the relationship between experimental data, clinical information, including the duration of illness (DOI), and the estimated total dosage of neuroleptics or anticholinergics prescribed.

### Spray coating of the matrix solution for IMS

A 2,5-dihydroxybenzoic acid (DHB) solution (40 mg/mL DHB, 20 mM potassium acetate, 70% Methanol, 0.1% trifluoroacetic acid) and a 9-aminoacridine solution (10 mg/mL, dissolved in 70% methanol) were used for imaging lipids. Methanol, potassium acetate, and ultra-pure water were purchased from Wako Chemicals (Osaka, Japan), and calibration-standard peptide and DHB were purchased from Bruker Daltonics. 9-aminoacridine was purchased from Acros (Pittsburgh, USA). 1-Palmitoyl-2-oleoyl-sn-glycero-3-phosphate was purchased from Funakoshi Co., Ltd. (Tokyo, Japan). All chemicals were of the highest purity available. The matrix solutions were sprayed over the tissue surface using a 0.2-mm nozzle caliber airbrush (Procon Boy FWA Platinum; Mr. Hobby, Tokyo, Japan). Sections to be compared were simultaneously spray-coated with each matrix solution to equalize analyte extraction and co-crystallization conditions. The distance between the nozzle tips and the tissue surface was 10 cm, and the spraying period was fixed at 5 min. Approximately 100 μL of matrix solutions were sprayed onto each brain section.

### IMS conditions

A MALDI TOF/TOF-type instrument (Ultraflex II TOF/TOF; Bruker Daltonics) was used for single MS imaging. The instrument was equipped with a 355-nm Nd:YAG laser. The data were acquired in the positive and negative reflectron modes under an accelerating potential of 20 kV using an external calibration method. Signals between *m/z* of 400 and 1000 were collected, and raster scans on tissue surfaces were performed automatically using FlexControl and FlexImaging 2.0 software (Bruker Daltonics). Laser irradiation consisted of 200 shots per spot. Image reconstructions were performed using FlexImaging 2.0 software. On-tissue molecular identification by MALDI-MS/MS was performed with a MS-IT-TOF (iMScope; Shimadzu Corporation, Kyoto, Japan) system. The molecular weight range for the ion trap was 1.0 Da around the *m/z* of the precursor ion. Laser irradiation consisted of 200 shots per spot at a 1000 Hz repetition rate. Mass spectra were obtained from a scanning mass range of 100–900 Da.

## Additional Information

**How to cite this article:** Matsumoto, J. *et al*. Decreased 16:0/20:4-phosphatidylinositol level in the post-mortem prefrontal cortex of elderly patients with schizophrenia. *Sci. Rep.* **7**, 45050; doi: 10.1038/srep45050 (2017).

**Publisher's note:** Springer Nature remains neutral with regard to jurisdictional claims in published maps and institutional affiliations.

## Figures and Tables

**Table 1:** Demographic information of schizophrenia and control subjects.

	Control	Schizophrenia
N	6	8
Male/Female	2/4	6/2
Age (hours)	80 (±8.0), n = 6	70 (±6.1), n = 8
PMI (hours)	19 (±24), n = 6	20 (±12), n = 8
DOI (years)	—	48 (±8.8), n = 8
Brain pH	6.0 (±0.36), n = 6	6.3 (±0.53), n = 8
Freezer storage time (years)	13 (±3.4), n = 6	8.4 (±3.3), n = 8
Estimated total dosage of neuroleptics (g)	—	17.0 (±16.3), n = 8
Estimated total dosage of anticholinergics (g)	—	3.75 (±4.64), n = 8

Abbreviations: PMI, postmortem interval; DOI, duration of illness. Note: Numeric data under age at death, PMI, DOI, and Estimated total dosage of drugs were mean values (standard deviations). Estimated total dosage = DOI × dose of drugs (3 months before death), neuroleptics represent chlorpromazine-equivalent dose, anticholinergics represent promethazine-equivalent dose.

**Table 2:** Correlation of clinical profiles and intensity of 16:0/20:4-PI and 18:0/22:6-PS in prefrontal cortex from patients with schizophrenia.

	DOI (years), n = 8	Estimated total dosage of neuroleptics, n = 8	Estimated total dosage of anticholinergics, n = 8
16:0/20:4-PI	*P* = 0.36 (r = 0.38)	*P* = 0.082 (r = −0.65)	*P* = 0.32 (r = −0.41)
18:0/22:6-PS	*P* = 0.44 (r = 0.32)	*P* = 0.93 (r = −0.038)	*P* = 0.53 (r = −0.26)

Abbreviations: DOI, duration of illness.

**Table 3:** SRM mode conditions.

PCs	Q1 (*m/z*)	Q3 (*m/z*)	CE (eV)	LPEs	Q1 (*m/z*)	Q3 (*m/z*)	CE (eV)
28:0 (IS)	722.4	662.4	25	16:0	452.3	196.1	45
32:0	778.6	718.6	25	18:0	480.3	196.1	45
32:1	776.4	716.4	25	18:1	478.3	196.1	45
34:1	804.6	744.6	25	20:4	500.3	196.1	45
34:2	802.6	742.6	25	22:4	528.3	196.1	45
36:1	832.6	772.6	25	22:6	524.2	196.1	45
36:2	830.6	770.6	25	p16:0	436.3	196.1	45
36:4	826.6	766.6	25	p18:0	464.3	196.1	45
38:4	854.6	794.6	25	PIs	Q1 (*m/z*)	Q3 (*m/z*)	CE (eV)
38:6	850.6	790.6	25	32:0	809.5	241.1	52
40:6	878.6	818.6	25	32:1	807.5	241.1	52
LPCs	Q1 (m/z)	Q3 (m/z)	CE (eV)	34:0	837.5	241.1	52
16:0	540.3	480.3	25	34:1	835.5	241.1	52
18:0	568.4	508.4	25	34:2	833.5	241.1	52
18:1	566.3	506.3	25	36:0	865.6	241.1	52
20:4	588.3	528.3	25	36:1	863.6	241.1	52
22:6	612.3	552.3	25	36:2	861.5	241.1	52
PEs	Q1 (*m/z*)	Q3 (*m/z*)	CE (eV)	36:3	859.5	241.1	52
34:1	716.5	196.1	45	36:4	857.4	241.1	52
36:1	744.6	196.1	45	38:3	887.6	241.1	52
36:2	742.5	196.1	45	38:4	885.5	241.1	52
36:4	738.5	196.1	45	38:5	883.5	241.1	52
38:4	766.5	196.1	45	38:6	881.5	241.1	52
38:5	764.5	196.1	45	40:4	913.6	241.1	52
38:6	762.4	196.1	45	40:5	911.6	241.1	52
40:4	794.6	196.1	45	40:6	909.5	241.1	52
40:5	792.6	196.1	45	LPIs	Q1 (*m/z*)	Q3 (*m/z*)	CE (eV)
40:6	790.4	196.1	45	16:0	571.3	241.1	52
p34:1	700.5	196.1	45	18:0	599.3	241.1	52
p36:4	722.6	196.1	45	18:1	597.3	241.1	52
p38:4	750.5	196.1	45	20:3	621.3	241.1	52
p38:5	748.5	196.1	45	20:4	619.3	241.1	52
p40:6	774.6	196.1	45	22:6	568.5	241.1	52
p40:7	772.5	196.1	45				

Abbreviations: Q1, First quadrupole; Q3, Third quadrupole; p, plasmalogen PE.
